# 3D Facial Pain Expression for a Care Training Assistant Robot in an Elderly Care Education Environment

**DOI:** 10.3389/frobt.2021.632015

**Published:** 2021-04-29

**Authors:** Miran Lee, Dinh Tuan Tran, Joo-Ho Lee

**Affiliations:** ^1^ Advanced Intelligent System Laboratory, Graduate School of Information Science and Engineering, Ritsumeikan University, Shiga, Japan; ^2^ Advanced Intelligent System Laboratory, Faculty of Information Science and Engineering, Ritsumeikan University, Shiga, Japan

**Keywords:** elderly care training robot, social robot, human-centered robotics, sensor systems for elderly care training, pain expression, care training

## Abstract

As the elderly population increases, the importance of the caregiver’s role in the quality of life of the elderly has increased. To achieve effective feedback in terms of care and nursing education, it is important to design a robot that can express emotions or feel pain like an actual human through visual-based feedback. This study proposes a care training assistant robot (CaTARo) system with 3D facial pain expression that simulates an elderly person for improving the skills of workers in elderly care. First, in order to develop an accurate and efficient system for elderly care training, this study introduces a fuzzy logic–based care training evaluation method that can calculate the pain level of a robot for giving the feedback. Elderly caregivers and trainees performed the range of motion exercise using the proposed CaTARo. We obtained quantitative data from CaTARo, and the pain level was calculated by combining four key parameters using the fuzzy logic method. Second, we developed a 3D facial avatar for use in CaTARo that is capable of expressing pain based on the UNBC-McMaster Pain Shoulder Archive, and we then generated four pain groups with respect to the pain level. To mimic the conditions for care training with actual humans, we designed the system to provide pain feedback based on the opinions of experts. The pain feedback was expressed in real time by using a projector and a 3D facial mask during care training. The results of the study confirmed the feasibility of utilizing a care training robot with pain expression for elderly care training, and it is concluded that the proposed approach may be used to improve caregiving and nursing skills upon further research.

## 1 Introduction

Students who wish to become caregivers can receive the necessary education at a medical institute for a specific amount of time to improve their care skills. They can accumulate experience using traditional methods such as watching videos, reading books, using medical mannequins (Aung et al., 2012), and role-playing with one another to simulate the care of an elderly patient. Practical clinical training is the most effective technique to help improve a caregiver’s skills. However, there is a risk and potential for injuries to elderly people with weakened muscles or joints when an unskilled caregiver performs a task for them. In addition, it is difficult to iteratively recruit elderly participants for care education, and elderly participants may experience fatigue or boredom due to repeated tests. Therefore, it is necessary to develop an effective care training robot that can mimic the behavior or symptom of the elderly and to enable caregivers to practice elderly care skills. Such a simulator robot can be used for care training on how to take care of elderly persons and for helping therapists to learn how to rehabilitate patients (Wang et al., 2012). In universities or institutes, simulator robots play an important role in teaching students how to evaluate clinical symptoms and diseases related to the muscle and joint (Diener and Hobbs, 2012; Mahoney et al., 2013).

### 1.1 Simulated Robot

**FIGURE 1:**
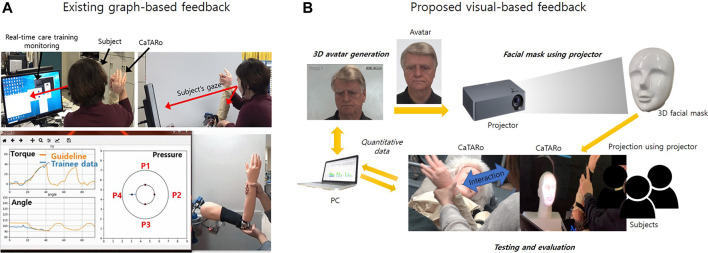
The proposed care training system with visual-based feedback.

The simulated robot consists of active and passive joints. Depending on the disease and the purpose of the target, there may be differences in the utilization of the motor. Active joints are used by the robot to move the joint by itself or to react to users’ actions. In contrast, passive joints allow the users to passively move the robot’s joints. A simulated robot imitates an actual human’s behaviors and activities. It can be used for improving care or nursing skills in interactions with care receivers. Matsumoto et al. (Matsumoto et al., 2018) described the development of an elderly whole-body robotic simulator for evaluating nursing care. Although several previous studies did not involve an elderly simulator robot per se, related simulator robots have been introduced. Fujisawa et al. (Fujisawa et al., 2007) proposed a human upper limb simulator for practical training experience; this simulator can reproduce the stiffness of an elbow joint to allow the trainee to improve their physical therapy stretching techniques. Mouri et al. (Mouri et al., 2007) developed a robot hand with symptoms of disability (contracture) for rehabilitation therapist education. In their system, distributed tactile sensors are used to estimate the elbow joint torque of the robot hand. Wang et al. (Wang et al., 2012; Wang et al., 2013) proposed a robotic arm for neurological examination training. Although these examples show that greater attention is being paid to patient simulator robots, the availability of simulators for human care training is still insufficient. It is also noted that we can take advantage of advances in disease-focused robot studies to further develop human simulator robots for care and therapy training. In addition, although simulated robots have been developed in many studies, the development of human–robot interaction system in which simulated robots can directly interact with humans is still lacking, as shown in Table 1. The human-simulated robot interaction for care education can be developed based on text-, alarm-, voice-, and visual-based methods. However, the simulated robot for care or nursing education still relies on the post-evaluation using statistical analysis, and a more advanced feedback method is required for interaction between users and robots. Particularly, the robust methods for robots to provide feedback to learners can be based on visual information and sounds. Huang et al. (Huang et al., 2014a; Huang et al., 2014b; Huang et al., 2015; Huang et al., 2016; Huang et al., 2017) proposed a robot for transfer training based on a voice feedback system that was used to enable the robot to receive nurses’ commands. Further, in our previous studies (Murata et al., 2017; Lee et al., 2019a; Lee et al., 2019b; Lee et al., 2020a; Lee et al., 2020b), a graph-based care education program was developed, and this method was designed as a program with built-in guidance of experts (caregivers) and helps users to learn care skills using simulated robots based on the graphs displayed on the monitor. However, the existing graph-based method has the following limitations: it is difficult to recognize the emotional state of the simulated robot and it is difficult to receive feedback on the pain or stress the robot feels during care education, as shown in Figure 1. Therefore, a visual-based feedback method is required to ensure amicable interactions between simulated robots and users.

**TABLE 1:** Comparison between the related work and the method proposed in this work.

References	Subject	Description	Feedback method
Matsumoto et al. (2018)	Elderly	Method to reduce the workload of caregivers through non-wearable transferring	Not provided
Fujisawa et al. (2007)	Patient	Upper limb simulator to reproduce the stiffness of spasticity	Not provided
Mouri et al. (2007)	Patient	Human hand with a disability for rehabilitation training	Not provided
Wang et al. (2013)	Patient	Robot for neurologic examination training	Not provided
Lin et al. (2019)	Patient	Robot for transfer training	Voice-based feedback statistical analysis
Huang et al. (2017)			
Huang et al. (2016)			
Huang et al. (2015)			
Huang et al. (2014a)			
Huang et al. (2014b)			
Kitajima et al. (2014)			
Lee et al. (2019a)	Elderly	Elderly robot for care training	Graph-based monitoring feedback in real time
Lee et al. (2019b)			Statistical analysis
Lee et al. (2020a)			
Lee et al. (2020b)			
Murata et al. (2017)			
Present study	Elderly	Elderly robot for care training	Visual-based method (robot’s facial expression)

### 1.2 Visual-Based Feedback

In care education, the robust feedback methods that robots can provide to learners can be based on visual information and sounds. Visual-based feedback is the most effective method in terms of practice for caregivers. The caregivers should periodically investigate whether the patient is feeling pain or not. In particular, it is very important to observe painful expressions on the patient’s face because the elderly may experience burden in terms of communication with caregivers. Although a robot’s expression can be communicated in various ways, the method of using a projector, in particular, has the main advantage of low cost and easy to use. In addition, the visual-based feedback that may be obtained by using a projector is able to represent a variety of realistic facial expressions. Maejima et al. (Maejima et al., 2012) proposed a retro-projected 3D face system for a human–robot interface. Kuratate et al. (Kuratate et al., 2011; Kuratate et al., 2013) developed a life-size talking head robotic system (Mask-bot) using a portable projector. Pierce et al. (Pierce et al., 2012) improved the preliminary Mask-bot (Kuratate et al., 2011; Kuratate et al., 2013) by developing a head with a 3-DOF neck for research into human–robot interactions. Robot heads with a projector are able to easily and iteratively improve and modify issues associated with the software display algorithm. Therefore, in this study, we propose a 3D facial pain expression–based care training assistant robot for elderly care education to improve the skills of caregivers by providing the visual-based feedback using a projector.

### 1.3 Passive Range of Motion

ROM, passive range of motion, exercise describes physical exercise that is used for improving the movement of joints. This ROM exercise is characterized by the musculoskeletal actions when performing an exercise (United Nations, 2013). The elderly may experience the limited movement of muscles and joints in daily life due to the symptoms such as stiffness, contraction, or weakness of muscles and joints. Therefore, caregivers or therapists must periodically ask elderly persons to do ROM exercise. However, it can be difficult for students or novices to gain care experience and skill in performing ROM exercises with a real human. Thus, we propose a care training assistant robot that allows trainees to effectively practice ROM exercises, as well as to receive the visual-based feedback on their care training skill.

### 1.4 Objective

As mentioned previously, elderly people frequently experience pain in the movement of joints during daily life; therefore, a caregiver should periodically help elderly patients to stretch and exercise. In this study, we propose a care training assistant robot (CaTARo) with 3D facial pain expression for improving care skills. This system is able to simulate the reduced physical movement of an elderly person for training while providing feedback on trainee performance. To evaluate this system, elderly care experts and trainees perform ROM exercises using the proposed CaTARo system. Because quality elderly care training experience is important, we have established the following requirements for our proposed CaTARo system:

1) It must imitate the joints of an elderly person and perform realistic joint movement.
2) It must reproduce the medical symptoms of elderly specific problematic joint movements.
3) It must provide appropriate quantitative assessment feedback on care training to the caregiver.
4) Using CaTARo, it must provide precise feedback to the trainee by comparing the trainee performance data with the data from an expert.

A preliminary version of this work has been introduced (Murata et al., 2017; Lee et al., 2019a; Lee et al., 2020b); in this study, we provide additional functions that can provide the feedback on the practical skill with 3D facial pain expression compared to the previous work. In addition, this study proposes a visual-based feedback method to ensure amicable interactions between simulated robots and users. The contributions of this study are as follows:

• A system that can effectively improve care or nursing skills of students is proposed by developing a simulated robot with more functions than those of existing methods such as books, videos, and medical mannequins.
• The proposed visual-based feedback method can improve the interaction skills between the users and the simulated robot.
• Students can receive feedback on the pain or stress state that a simulated robot may feel in a real-time environment.
• Based on the proposed visual-based feedback method, students can respond immediately to the pain experienced by simulated robots in a real-time environment.

To generate the 3D facial avatar of CaTARo, we referred to the method for measuring the pain level from facial images (Prkachin, 1992; Lucey et al., 2011). In the study (Lucey et al., 2011), the facial images of subjects were acquired during shoulder ROM exercises, and the pain levels were obtained from the images by the pain level measurement method derived by Prkachin and Solomon (Prkachin, 1992). In this study, however, we determine four pain groups with respect to the pain level from facial images of the UNBC-McMaster Pain Shoulder Archive database (Lucey et al., 2011). The facial images consistent with the range of each pain group are extracted and used as representative images of the 3D avatar of CaTARo.

## 2 Care Training Assistant Robot

**FIGURE 2:**
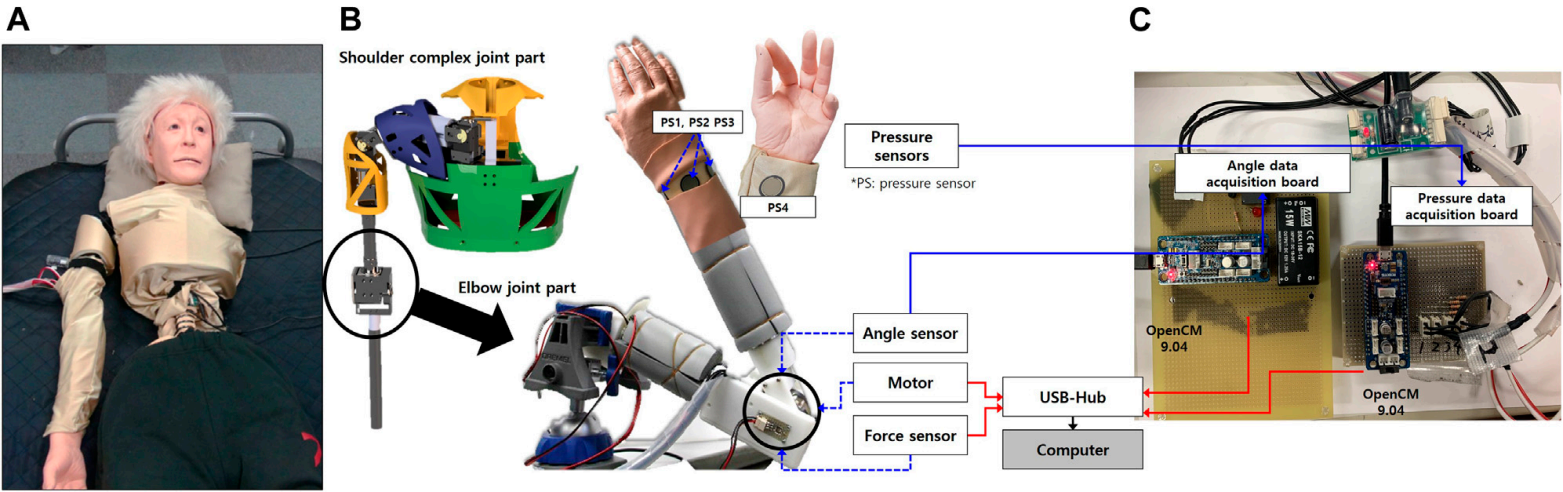
Care training assistant robot (CaTARo): **(A)** prototype of the upper limb, **(B)** 3D modeling of upper limb, and **(C)** hardware configuration of the elbow part.

Passive ROM exercise is a type of physical exercise that is used for improving the movement of joints, which is one of the most important tasks in elderly care because the elderly may experience limited joint movement in daily life due to the stiffness, contraction, or weakness of muscles. Considering a care training robot, we propose the use of CaTARo, which was developed as a method to effectively improve the care abilities of caregivers or students in preliminary studies (Murata et al., 2017; Lee et al., 2019a; Lee et al., 2020b). As shown in Figure 2, the upper limb of CaTARo consists of the glenohumeral, sternoclavicular, and elbow joint based on six degrees of freedom (DOF). In this study, we used the elbow joint of CaTARo to evaluate the feasibility of our proposed system.

### 2.1 Hardware Components and Configuration

More detailed explanations of the hardware design and the way in which CaTARo reproduces the joint angle and torque of the elderly are described in (Murata et al., 2017; Lee et al., 2019a; Lee et al., 2020b). In brief, according to the Human Body Properties Database (NITE, 2019 accessed on 2019–05–06) in Japan, the statistics show that elderly people had the average upper arm and forearm lengths of 289 and 225 mm, respectively, and the average circumferences of the upper arm and forearm were 280 and 242 mm, respectively. Based on these statistical data for real humans, we designed the elbow joint of CaTARo with an upper arm length of 280 mm, a forearm length of 220 mm, an upper arm circumference of 285 mm, and a forearm circumference of 240 mm.

As shown in Figure 2B, the elbow joint of CaTARo consists of four main components: a servo motor (MX-28, Robotics Inc., Seoul, South Korea), an angle sensor (SV01L103AEA11T00, Murata Electronics Co. Ltd., Kyoto, Japan), a force sensor (CFS018CA201U, Leptrino Inc., Nagano, Japan), and four pressure sensors (FlexiForce A201, Tekscan, Inc., MA, United States). The robot is covered with artificial skin such that it can appear more human-like, which increases its authenticity for the trainee. Figure 2C shows the hardware configuration. The elbow actuator angle and four pressure sensors embedded in the CaTARo are obtained through custom hardware boards and then transmitted to a computer. The torque data from force sensor are transmitted to the computer. The sampling rate of all sensors is set to 100 Hz.

### 2.2 Care Training Monitoring Program

To acquire the angle, torque, and pressure values from CaTARo, a custom care training monitoring program was developed using Python. The elbow joint of CaTARo is connected to the computer *via* serial communication, and the data are input simultaneously into the computer. The guideline indicated in the graph is obtained from an elderly care expert in advance, and the student performs care training based on the expert’s guideline in real time.

## 3 3D Facial Pain Expression With CaTARo

In this section, the 3D avatar facial pain expression of CaTARo is generated according to the level of pain in the image to express pain. The method used to generate the 3D avatar of CaTARo is divided into three stages: 1) measurement of the pain level using CaTARo, 2) extraction of the pain expression level from facial images, and 3) 3D facial expression and projection. First, we measure the pain level that a trainee has given to CaTARo in care education. Next, we extract an image which contains pain expression from facial images that have pain expressions by referring to the method derived from the study (Lucey et al., 2011). The pain expression image can be divided into pain groups (PGs) of four stages to select images representing each group, and avatars are then generated based on the images. Finally, the avatars of the four produced groups are projected in the 3D facial mask *via* a projector.

### 3.1 Pain Level Based on Fuzzy Logic

The pain level is a quantitative value that can be used to assess a student’s ability to train care’s skill without burdening the joint of CaTARo during care education. We used fuzzy logic to measure the pain level, and two hypotheses were incorporated to generate the fuzzy logic model.

• The resistance torque increases when the elbow joint of CaTARo is below 90° or above 120°. The range of motion of CaTARo’s joint was designed based on expert opinion to mimic the joint of an elderly person. If the joint angle of CaTARo is outside the range of 90–120°, CaTARo is assumed to feel “pain.”
• The reference data for generating membership functions are based on data from five trials involving an expert who has years (two to ten years) of experience in the medical field and care of elderly persons. In future studies, the reference data may include data from elderly persons or data from elderly persons with joint or neurological diseases.

$$f_{s}\left(X\right)=\frac{\sum_{l=1}^{M}\theta_{l}{\prod^{\text{​}}}_{k=1}^{P}\mu_{F_{k}^{l}}\left(x_{k}\right)}{\sum_{l=1}^{M}{\prod^{\text{​}}}_{k=1}^{P}\mu_{F_{k}^{l}}\left(x_{k}\right)}.$$ (1)

The fuzzy set theory of Zadeh Zadeh (1973) is frequently used as a suitable method to consider ambiguous problems that are complex or uncertain in real-world contexts. A fuzzy system has the advances that it determines the relationships between input and output variables and it can describe the interpretation of relationships among input variables (Kumar et al., 2007). The output values of the fuzzy logic method can be obtained by using Eq. 1, Where *x*, *k*, *M*, and *P* indicate the input variable, the *k*th element of the vector *x*, the number of membership functions, and features, respectively. $\mu_{F_{k}^{l}}\left(x_{k}\right)$ is the membership function and $\theta_{l}$ is the weight.

Input variables of the angle, angular velocity, torque, and the mean of the signal magnitude area of pressure values (pressure) were extracted to generate fuzzy logic modeling. The input variables were divided into three groups as “low-immoderate,” “moderate,” and “high-immoderate.” The input variable pressure and output variable “pain level” consist of “low,” “medium,” and “high.”

**FIGURE 3:**
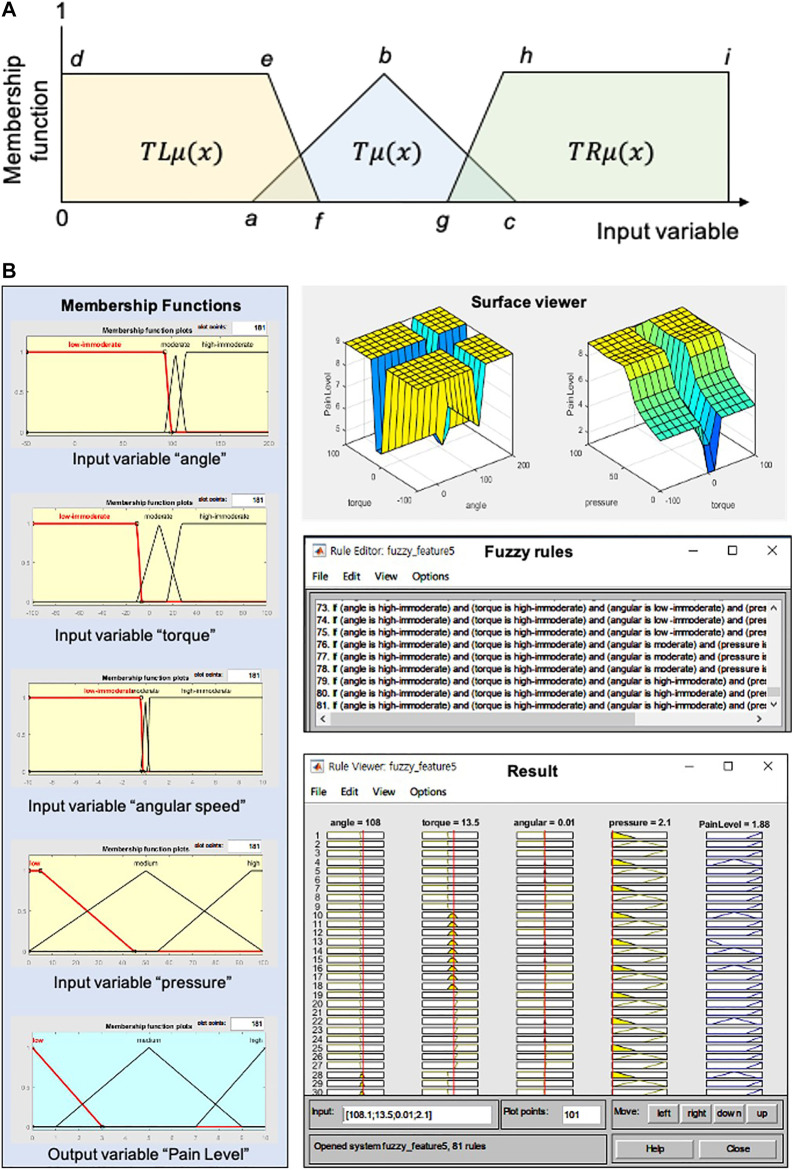
Fuzzy logic method for the pain level: **(A)** fuzzy membership functions for four input variables (angle, torque, angular velocity, and SMAP) and an output variable (pain level), surface view of input variables, 81 fuzzy rules, and result. SMAP means the signal magnitude area of pressure values; **(B)** fuzzy membership functions for four input variables (angle, torque, angular velocity, and SMAP) and an output variable (pain level), surface view of input variables, 81 fuzzy rules, and result. SMAP means the signal magnitude area of pressure values.

As shown in Figure 3A, we used two trapezoidal membership functions (boundary variables) and one triangular membership function (intermediate variables). Three types of membership functions can be calculated using Eqs 2–4.

$$T\mu \left(x\right)=\{\begin{array}{cc} 0, & \left(x\leq a\right)or\;\left(x\geq c\right)\\ 1, & x\equiv b\\ \frac{x-a}{b-a}, & a\leq x\leq b\\ \frac{c-x}{c-b}, & b\leq x\leq c \end{array},$$ (2)

$$TL\mu \left(x\right)=\{\begin{array}{cc} 1, & d\leq x\leq e\\ \frac{f-x}{f-e}, & e\leq x\leq f\\ 0, & x\geq f \end{array},$$ (3)

$$TR\mu \left(x\right)=\{\begin{array}{cc} 0, & x\leq g\\ \frac{h-x}{h-g}, & g\leq x\leq h\\ 1, & h\leq x\leq i \end{array},$$ (4)

where *x* is the input variable. Tμ(x), TLμ(x), and TRμ(x) indicate the triangular, left-trapezoidal, and right-trapezoidal membership functions, respectively. The parameters *a* to *f* refer to the constants of the input variables that determine the value of the membership function. In this study, a trapezoidal membership function on the left and right for the angle and torque input parameters was used. The reason for this implementation is that CaTARo has a limited range of motion in order to reproduce the joint motion of the elderly. The range in which CaTARo feels pain is defined based on the care training assessment factor in Table 2. The trapezoidal membership function reflects the fact that CaTARo receives the maximum pain when it is out of the range of joint motion. Therefore, a trapezoidal membership function was applied to set the weight of the membership function as “1” in the range where CaTARo feels pain.

**TABLE 2:** Care training quantitative assessment factors.

Factors	Parameters	Values
Angle (degree)	Low	100
	Moderate	95 to 125
	High	110
Torque (Nm)	Low	-10
	Moderate	−15 to 35
	High	30
Angular velocity (degree/s)	Low	−1
	Moderate	−1.5 to 1.5
	High	1
SMA of pressure values	Low	40
	Moderate	20 to 80
	High	60

Referring to Table 2, the parameters such as *a*, *b*, *d*, *f*, and *g* were calculated by using Eqs 5–7. The parameters *a* and *e* can be obtained by the difference between the mean and minimum values of the factors, while the parameters *c* and *h* can be calculated as the sum of the difference between the mean and maximum values of the factors. The parameter *b* is determined by the mean value of the parameters *a* and *c*. The parameters *d* and *i* indicate the minimum and maximum angle values allowed by the joint of CaTARo, respectively. In the triangular membership function Tμ(x), the parameter *f* is obtained by the sum of its deviation from the mean of minimum value of factors, while the parameter denoted as *g* can be calculated as the difference of its deviation from the mean of the maximum value of factors.

$$\begin{array}{c} a=e=MNP-\sqrt{\frac{{\sum_{k=1}^{n}\left(param_{k}-param_{m}\right)}^{2}}{n}} \end{array},$$ (5)

$$\begin{array}{c} f=g=MXP+\sqrt{\frac{{\sum_{k=1}^{n}\left(param_{k}-param_{m}\right)}^{2}}{n}} \end{array},$$ (6)

$$\begin{array}{c} b=\left(\left|f|-|c\right|\right)/2 \end{array},$$ (7)

where $param_{k}$ and $param_{m}$ denote that the *k*th input value of parameter and the mean of input parameter value, respectively.

The next step is generating fuzzy rules that define the relationships among the input variables. The fuzzy rules were constructed using an expert’s opinion and can be described using Eq. 8:

$$rule^{k}:IF\;X_{k}\;is\;F_{1}^{k}\;and\ldots and\;X_{p}\;is\;F_{p}^{k}\;then\;Y^{k},$$ (8)

where $F_{1}^{k}$ is the *p*th fuzzy set associated with the *k*th rule. The rule “IF $x_{k}$ is $F_{1}^{k}$ and ⋯ and $X_{p}$ is $F_{p}^{k}$” corresponds to the antecedent from the *k*th rule and “THEN *Y* is $Y^{k}$” is the consequent of the *k*th rule.

Finally, a total of 81 fuzzy rules were generated, as shown in Figure 3B (right-bottom). The number of input variables is four (angle, torque, angular velocity, and pressure), and the membership functions consist of three kinds of antecedent variables (low, medium, and high). The number of fuzzy rules can then be calculated as $m^{n}$ (*m* and *n* indicate the numbers of input and antecedent variables, respectively). Therefore, there were 81 rules = (3 × 3 × 3 × 3) that were each connected with one of the three antecedent variables for the output (pain level).

In this study, we determine the inference engine using the Mamdani method to design the fuzzy inference system (FIS). For FIS, there are two main types of methods of mapping inputs to outputs: Mamdani FIS and Sugeno FIS. The Mamdani-based FIS has the advantages of being intuitive, well-suited to human inputs, more interpretable fuzzy rule, and applicable in various fields such as medical diagnostics, industrial manufacturing, hospitals, and banks (Gayathri and Sumathi, 2015). Next, defuzzification is performed, in which the output of the inference engine is calculated to a crisp value; the defuzzifier is an important component of FIS (Ahmad et al., 2019). Since the centroid defuzzification method is the most commonly and frequently used (Gayathri and Sumathi, 2015) and has a robust performance, it was used as the final diffusion layer in this study. Finally, the pain level output obtained by the input variables ranged from 0 to 10.

### 3.2 Extraction of a Facial Image With Pain Expression

**FIGURE 4:**
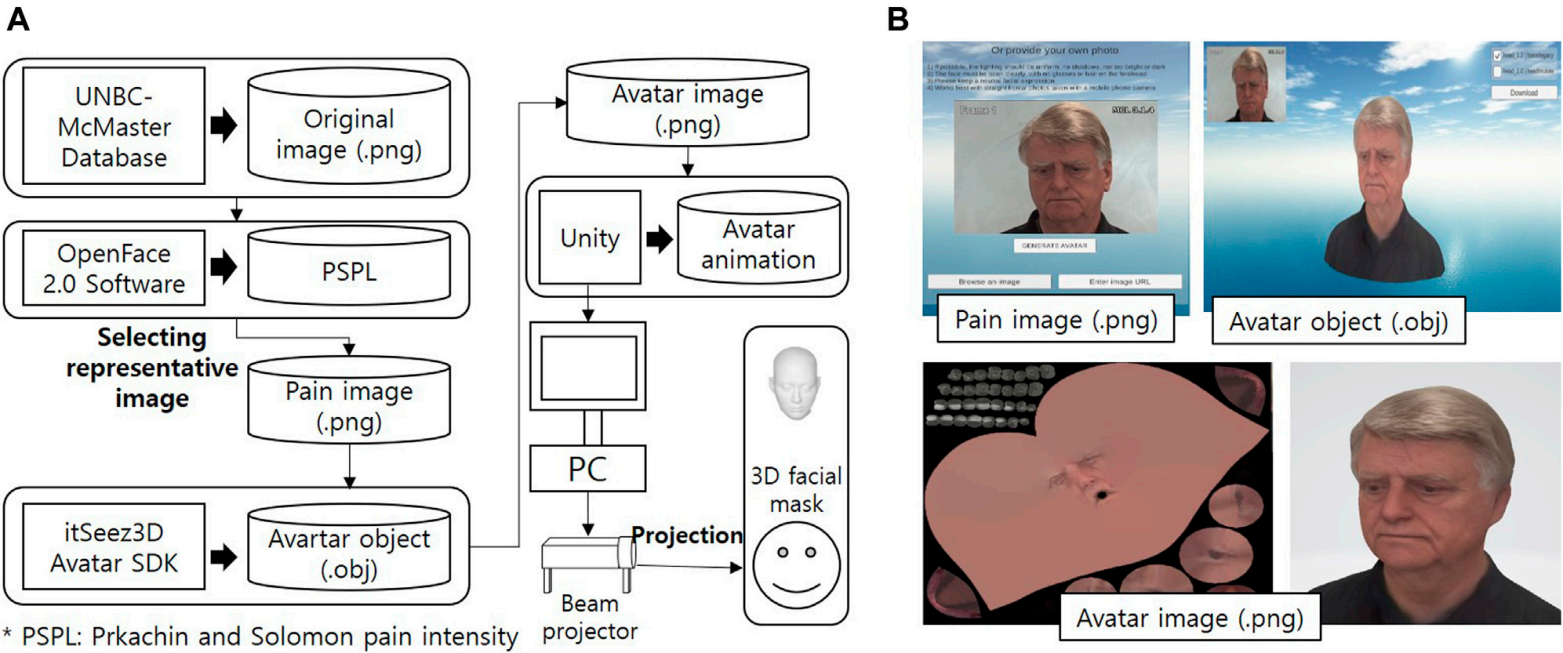
Framework of the proposed method in this study: **(A)** framework and **(B)** the method to generate the avatar image.

Figure 4A shows the framework of the 3D facial expression system of CaTARo. The original facial image for pain expression can be extracted by using FACS (Ekman et al., 2002). Among the action units (AUs) related to pain expression, six types of AUs including brow lowering (AU4), cheek-raising (AU6), eyelid tightening (AU7), nose wrinkling (AU9), upper-lip raising (AU10), and eye closure (AU43) can be used to calculate the pain level during passive ROM exercise (Prkachin, 1992; Lucey et al., 2011). The pain level, called PSPI (Prkachin and Solomon Pain Intensity Scale), can be found by using Eq. 9:

$$\begin{array}{c} PSPI=AU4+\left(AU6\;or\;AU7\right)+\left(AU9\;or\;AU10\right)+AU43 \end{array},$$ (9)

where (AU6 or AU7) and (AU9 or AU10) indicate that the higher intensity is chosen.

To generate the 3D facial avatar with pain expression for the CaTARo system, we used the public database UNBC-McMaster Pain Shoulder Archive (Lucey et al., 2011). This database consists of facial expressions from 129 participants who were suffering from shoulder pain (they performed different passive range of motion tests), and the PSPI values were extracted. Figure 4B shows the procedures for generating the 3D facial expressions of CaTARo using the UNBC-McMaster Pain Shoulder Archive. First, the values of each AU required to obtain the PSPI values were extracted using the OpenFace 2.0 tool kit (Baltrusaitis et al., 2018).

**FIGURE 5:**
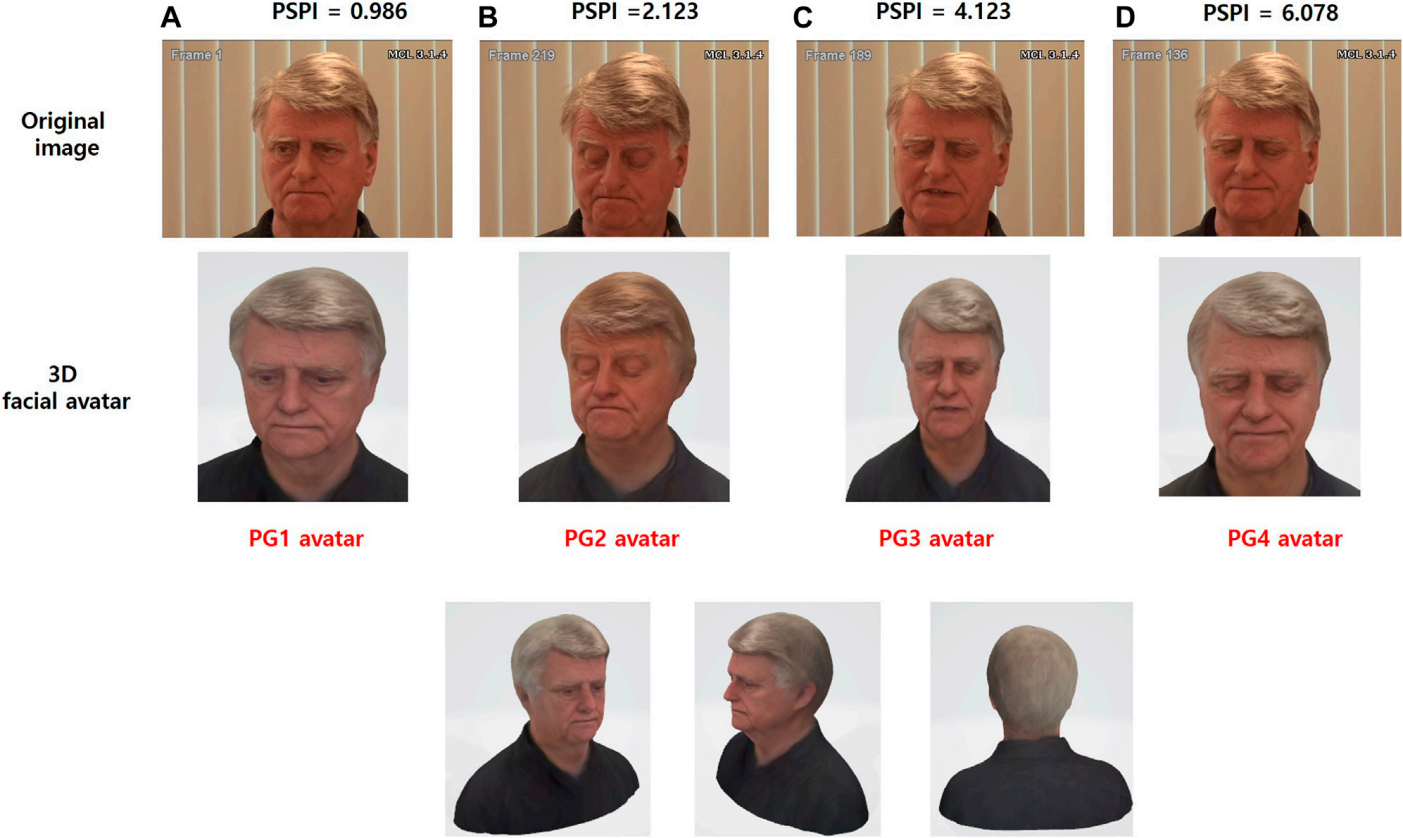
The procedures of generating 3D facial avatar using the Prkachin and Solomon pain intensity (PSPI) of subject #TV095 from the UNBC-McMaster Pain Shoulder Archive. **(A)** The PG1 avatar was generated based on the PSPI of 0.986 from the original image (frame #1). **(B)** The PG2 avatar was generated based on the PSPI of 2.213 from the original image (frame #219). **(C)** The PG3 avatar was generated based on the PSPI of 4.123 from the original image (frame #189). **(D)** The PG4 avatar was generated based on the PSPI of 6.078 from the original image (frame #136).

Table 3 shows the results of the final PSPI values and the values of the AUs obtained from four types of original images from subject #107 in the UNBC-McMaster database. In this study, the range of PSPI was divided into four groups. Pain group 1 (PG1) ranges from 0 to 1.99 PSPI, PSPI ranges from 2 to 3.99 as PG2, PG3 ranges from 4 to 6.99 PSPI, and PSPI ranges from 7 to 9.99 for PG4. The original images shown in Figures 5A–D show the example of procedures of generating for 3D facial avatar of subject #TV095 in the UNBC-McMaster database.

**TABLE 3:** Discrimination of pain group (PG) based on PSPI result of record #107 from UNBC-McMaster Pain Shoulder Archive.

	AU4	AU6 or AU7	AU9 or AU10	AU45	PSPI	PGs
Frame #1	0.14	0.56	0	1.24	1.94	PG1
Figure 5A						
Frame #2	0.61	0.71	0.83	1.11	3.25	PG2
Figure 5B						
Frame #3	1.51	1.76	0.23	1.83	5.33	PG3
Figure 5C						
Frame #4	1.35	3.42	1.83	1.12	7.72	PG4
Figure 5D						

PG means pain group; PG1: PSPI (0–1.99); PG2: PSPI (2–3.99); PG3: PSPI (4–6.99); PG4: PSPI (7–9.99).

### 3.3 3D Facial Expression and Projection

The original images, which were divided into four pain groups (PGs), are generated as 3D avatars using the Avatar SDK (Itseez3D, Inc., CA, United States). As shown in Figure 4B, the 3D avatars are converted from original images (.png) to avatar objects (.obj) using a web-based SDK. The Avatar SDK (Itseez3D, Inc., CA, United States) acts as the conversion SDK, and object files are acquired from the original image. The 3D avatar (.png) image can be converted when the object file is downloaded. After loading the generated 3D avatar, object files (.obj) are handled in the Unity program, and facial avatars with pain expressions for each of the four PGs were finally created, as shown in the bottom of Figure 5E. Finally, CaTARo and the 3D facial avatar are connected to the Unity program, and the avatar is projected onto the 3D facial mask *via* a projector (Figure 4A). The pain level calculated by the sensor value of CaTARo is calculated to determine the corresponding PG group, and the final PG avatar matching the pain level is projected.

## 4 Experimental Environment

### 4.1 Evaluation Objectives

The effect of using CaTARo in elderly care training was analyzed based on the following three evaluation objectives:

• In the care training monitoring-based feedback environment, were there any significant differences in care skills between experts and students?
• Is there any significant difference between the expert and student groups in the pain-level measurement result?
• Does CaTARo with the visual-based feedback provide a challenging feasibility in care education?

### 4.2 Participants

The experiments were conducted with nine subjects (five elderly care experts and four students). The elderly care experts (four female and one male) were caregivers who had been working in the rehabilitation and medical fields for over two years in Japan. The other four subjects (two male and two female) were students at Ritsumeikan University who had never experienced elderly care training and had never used CaTARo. All subjects agreed to participate in this experiment and signed a consent form, and the researchers worked to ensure their safety. The experiment was approved by the Institutional Review Board (IRB) of Ritsumeikan University (BKC-2018–059).

### 4.3 Experimental Design

**FIGURE 6:**
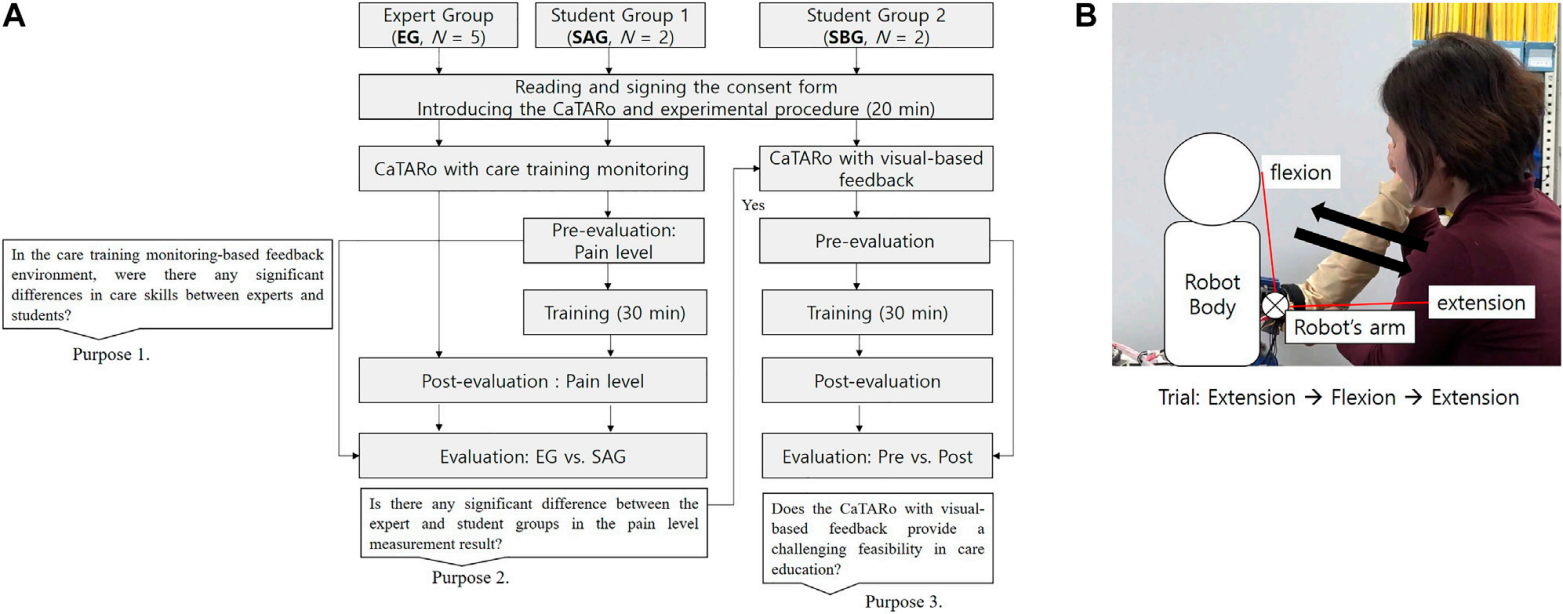
Experimental design and task procedure: **(A)** experimental procedure and **(B)** care task procedure.

Figure 6A presents the experimental procedure. Based on the evaluation objectives described in Section 4.1, the experiments were conducted in three groups as follows: 1) expert group (EG), 2) student group 1 (SAG), and 3) student group 2 (SBG). EG consisted of five experts and was used to evaluate the feasibility of the CaTARo system. To find the feasibility of CaTARo with visual-based feedback, only the participants in EG and SAG were provided quantitative data to evaluate the pain-level calculation results of CaTARo. In the evaluation between these two groups, we achieve two of the three objectives described in Section 4.1. Finally, in order to evaluate the feasibility of the visual-based CaTARo system, the participants in SBG conducted pre- and post-evaluation according to the task procedure (details are described in Section 4.4) using CaTARo with the visual-based method, as illustrated in Figure 6A.

### 4.4 Task Procedure

**FIGURE 7:**
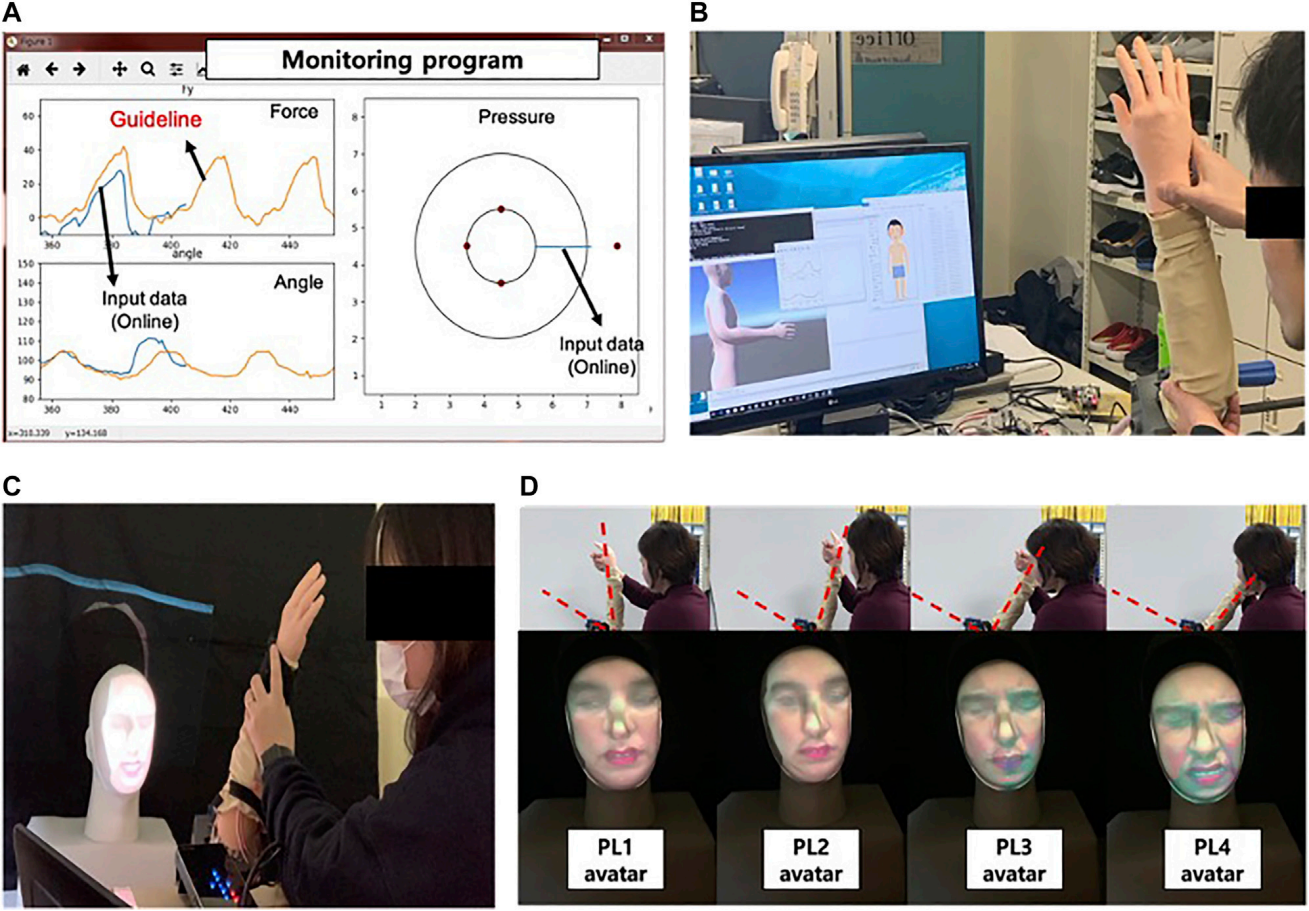
Experimental environment: **(A)** monitoring program: the orange graph on the left figure which represents the quantitative data from expert in advance. The students can receive the training based on the expert’s guideline and can experience the pain to feel the elderly through the circle graph appearing on the right screen. The pressure value on the right screen represents the magnitude of the pressure from a small circle to a large circle when pressure is detected, **(B)** experiment using CaTARo without 3D facial expression, **(C)** experiment using CaTARo with 3D facial expression, and **(D)** sample avatar output using beam projector with 3D facial mask.

To evaluate the proposed CaTARo system, a range of motion (ROM) exercise was performed. The ROM exercise is a type of care and rehabilitation activity which involves physical exercise for improving the movement of joints. The elderly may experience limited joint movement in their daily activities owing to a variety of musculoskeletal diseases such as weakness of muscles, joints, stiffness, and ligaments. Therefore, caregivers and therapists must periodically ask the elderly to perform the ROM exercise. In this study, the participants were asked to perform an ROM exercise based on elbow flexion and extension using the elbow joint of CaTARo. The experimental process is as follows. First, an expert performs ROM exercise using the elbow joint part of CaTARo, and the expert explains the ROM exercise to the students before starting the care training, and then, the students watch a video of the expert. In pre-evaluation, students perform the ROM exercise with the robot, and the quantitative data are obtained, and students practice the ROM exercise for 30 min using CaTARo on the practice system. For post-evaluation, students perform the ROM exercise in order to investigate the effectiveness of practice using CaTARo. As shown in Figure 6B, in total, 10 tasks were performed, and each task was performed for 10 trials per individual. Each individual took approximately 1 h for the experiment. Since care movements at the starting and cessation points may contain noise signals from the user’s movement, only data of five trials out of the total were used for evaluation. Figure 7 shows the experimental environment of the care training using the CaTARo system.

### 4.5 Evaluation Measure

To analyze the relationship among the results, the Mann–Whitney *U* test, two-sample *t*-test, and analysis of variance (ANOVA) method were used. Statistical analysis was performed using MATLAB 2019. The significance level α (=0.05) was commonly set. A single asterisk (*) indicates statistical significance at *p* < 0.05, and double asterisks (**) indicate statistical significance at *p* < 0.001.

## 5 Results and Discussions

### 5.1 CaTARo Using Graph-Based Feedback Method

**FIGURE 8:**
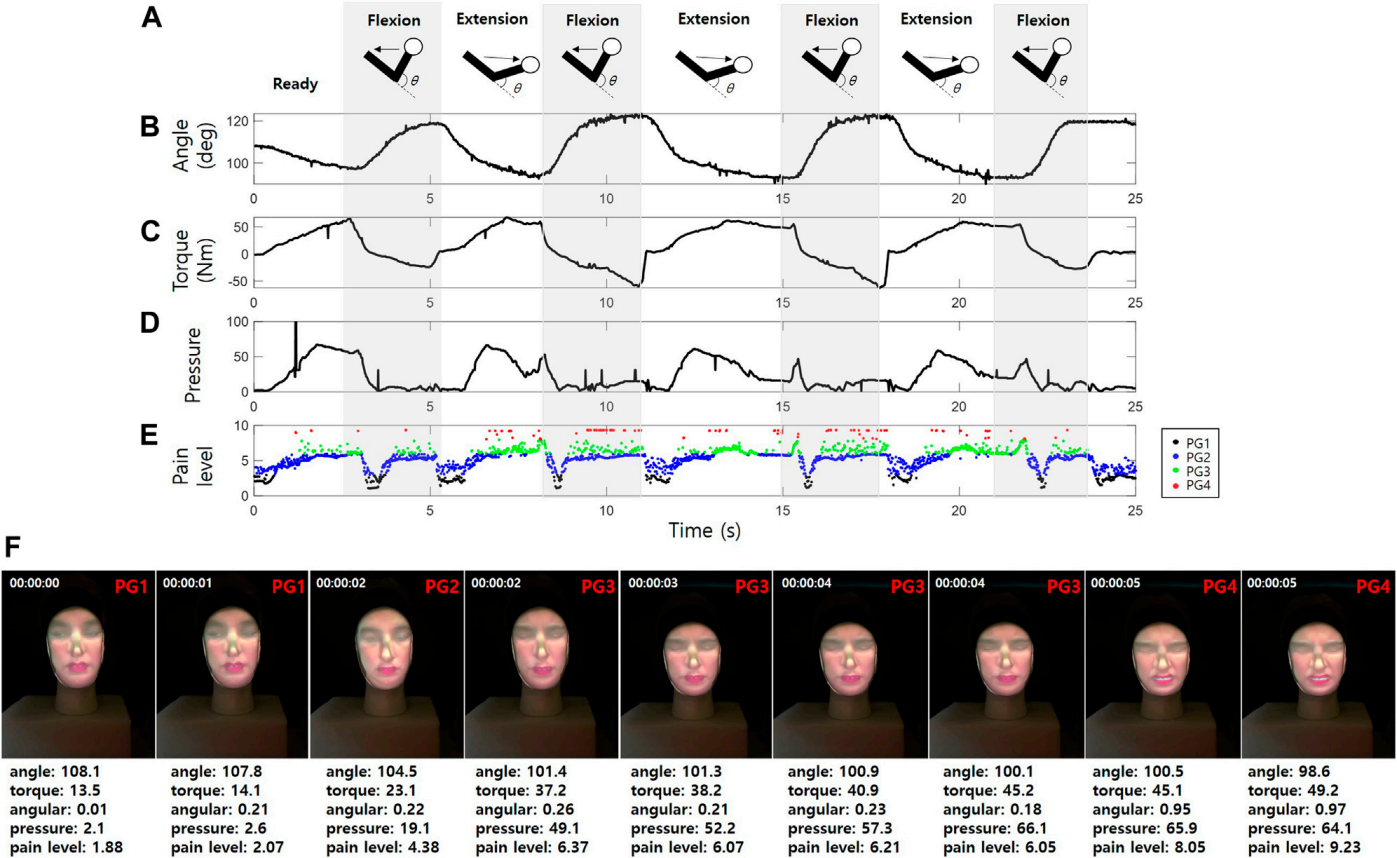
Example of quantitative data output from the elbow joint of CaTARo: **(A)** elbow kinematics, **(B)** elbow joint angle, **(C)** torque, **(D)** pressure data, **(E)** pain level extracted by fuzzy logic, and **(F)** testing of 3D facial pain expression with CaTARo.

To test the 3D facial expressions with the proposed CaTARo system, two subjects were chosen to participate in this experiment (SBG). Figure 8 depicts an example of the quantitative data (i.e., the elbow joint angle, torque, and pressure data) obtained from the output of the elbow joint of CaTARo and the pain level determined using the fuzzy logic method. Figure 8A shows the elbow kinematics of CaTARo; the torque and pressure data of the robot are simultaneously outputted when the elbow of the robot moves *via* extension and flexion. In Figures 8C,B, it can be seen that when the elbow joint angle of the robot decreases, the torque increases because the test subject has extended the elbow of the robot. Figure 8D presents the pressure data results, wherein the values alternate between the extension and flexion movements. These pressure sensor values are different for each participant because each participant may have applied different degrees of force and used different holding positions on the wrist of the robot. Figure 8E shows the pain level determined *via* fuzzy logic. The pain groups were categorized according to the pain level. In Figure 8E, the black dots (PG1), blue dots (PG2), green dots (PG3), and red dots (PG4) indicate the pain level ranges from 0 to 1.99, 2 to 3.99, 4 to 6.99, and 7 to 9.99, respectively. The results of the classified PG groups are projected onto the 3D facial mask in real time, as shown in Figure 8F.

**FIGURE 9:**
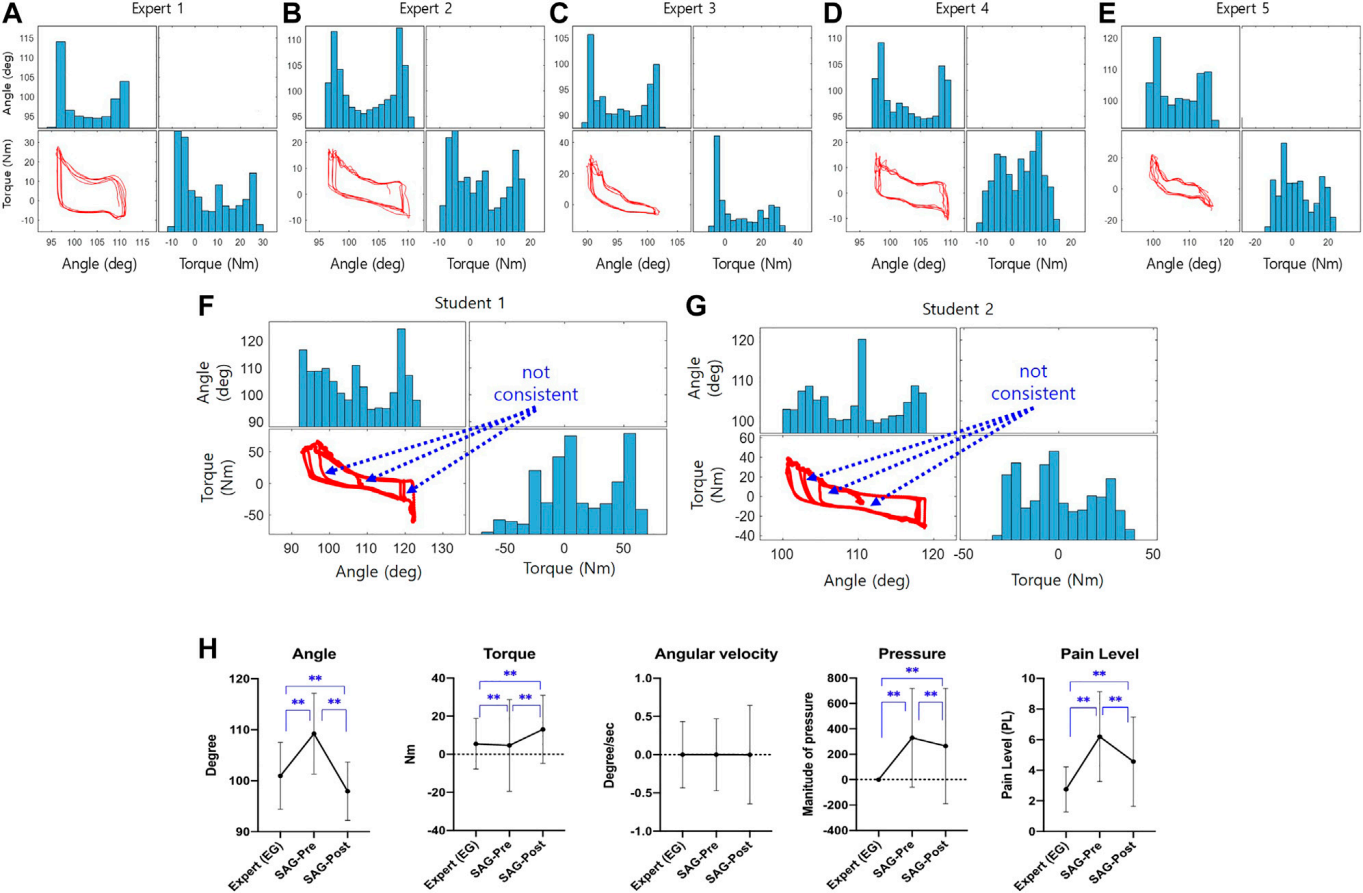
The result of graph-based method: **(A–G)** plot matrixes of between angle and torque of the robot obtained when experts perform the ROM exercise, and from **(A–E)** denotes the expert #1 to #5, respectively (The graphs of blue bar and red plot represent the histogram and plot between external angle and torque, respectively.); **(H)** comparison result of statistical analysis of parameters as angle, torque, angular velocity, pressure parameters, and pain level (EG: expert group; SAG: student group when using CaTARo with care training monitoring). *p 0.05 and **p 0.001.

Figure 9A to Figure 9E present scatterplot matrices based on the external angle and torque of CaTARo. These plots were obtained when the elderly care experts (EG) performed the specified ROM exercise using the robot. The histograms (the blue bars in Figure 9) of the elbow angle of CaTARo obtained by the experts using the training system show a tendency for the angle to decrease at the beginning of the ROM exercise (extension) and then increase at the ending. In addition, a similar pattern is noted from each expert participant when repeating the ROM exercise five times. This indicates that during the ROM exercise, the experts were more likely to repeat movement with a constant angle and speed of repetition. In contrast, as shown in Figures 9F,G, the results of the students (SAG) showed nonuniform angles and nonconstant durations during their five repetitions of the ROM exercise.

According to the ANOVA test analysis of the results obtained by the experts, the parameters of angle (*F* = 11.878, *p* = 0.165), angular velocity (*F* = 0.045, *p* = 0.996), and pressure (*F* = 1.325, *p* = 0.257) showed no significant differences among the experts, whereas torque values showed significant differences (*F* = 145.09, *p* < 0.001). Figure 9H shows the results of statistical analysis of the angle, torque, pressure, and fuzzy logic–based pain level in care training using CaTARo with care training monitoring performed by the experts (EG) and students (SAG) in pre- and posttests. The statistical analysis between the expert and the student group was performed using the Mann–Whitney *U* test method, and the statistical analysis of the pre- and post-evaluations between the student groups was conducted based on the *t*-test method. Particularly, the angle showed the most significant difference among these groups. The angle of SAG in the pretest (M = 109.23, SD = 7.93) before care training showed a large difference from that of EG (M = 100.97, SD = 6.55), and there was a significant difference (*p* < 0.01, Mann–Whitney *U* test). After care training for 30 min, the angle value of SAG (posttest) (M = 97.93, SD = 5.71) decreased to a similar range as that of EG, and there was a statistically significant difference (*p* < 0.01, Mann–Whitney *U* test). With respect to the torque, the posttest result of SAG (M = 13.02, SD = 17.87) was relatively higher than that of EG (M = 5.44, SD = 13.31), but it was confirmed that the standard deviation showed a relatively wide range in the pre-test (M = 4.61, SD = 24.08). For the torque, there was a statistically significant difference (*p* < 0.01, Mann–Whitney *U* test). In terms of the pressure parameter, EG (M = 0, SD = 0) showed values of practically zero, whereas the pressure values of SAG showed a significant difference (t = 8.045, *p* < 0.01) in both the pretest (M = 314.47, SD = 257.37) and posttest (M = 260.07, SD = 130.12). As indicated by the results of the final calculated pain level based on quantitative data (angle, torque, angular velocity, and pressure), the pain level in the pretest of SAG (M = 6.21, SD = 2.94) increased compared to EG (M = 2.76, SD = 1.47), and there was a statistically significant difference (*p* < 0.01, Mann–Whitney *U* test). The pain level decreased again in the post-evaluation after 30 min of care education (M = 4.57, SD = 2.92). Consequently, except for the parameter of angular velocity, statistical analysis was performed among the three groups, and all results showed significant differences. However, the results of care training for SAG showed a relatively similar range of movement to that of EG in the posttest than in the pretest, and it was confirmed that the training skills of the SAG participants were relatively improved.

### 5.2 Pilot Testing of CaTARo Using the Visual-Based Feedback Method

**FIGURE 10:**
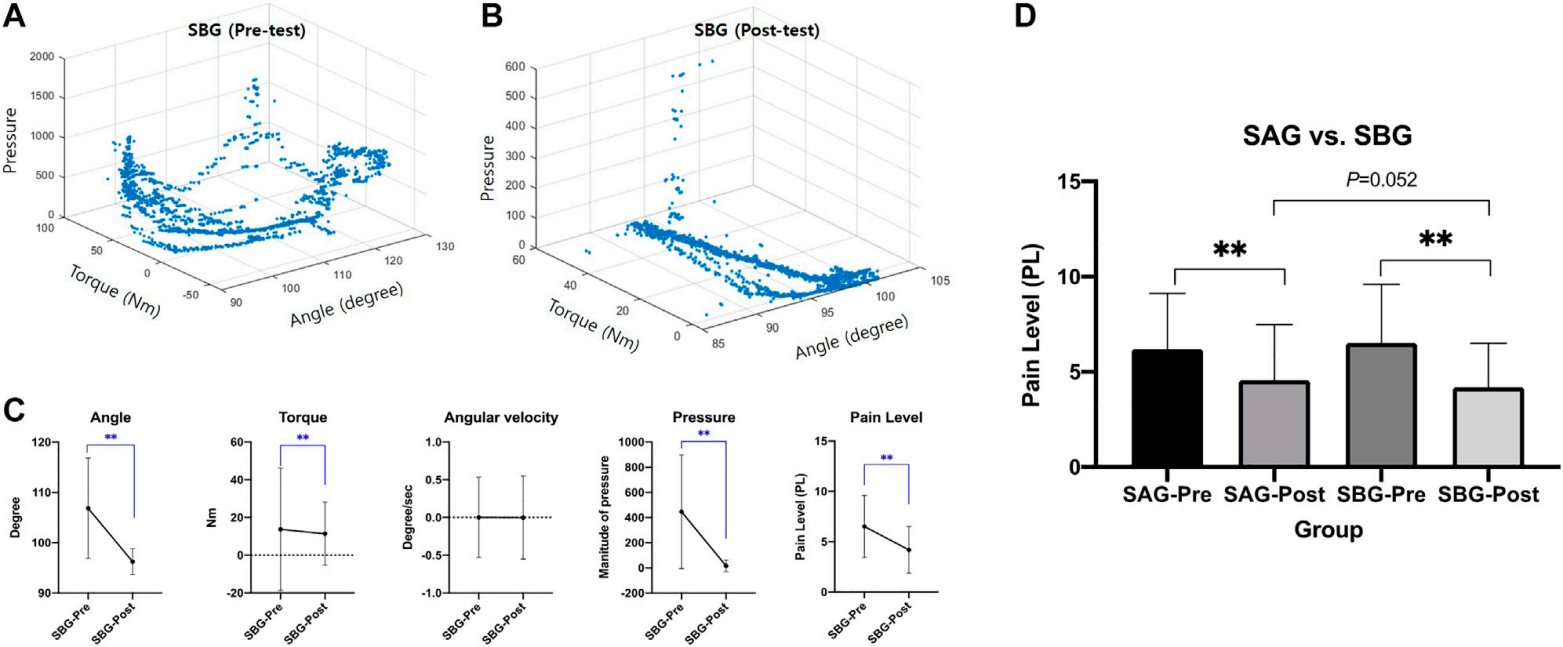
Results of CaTARo with visual-based feedback: **(A, B)** are the examples of scatterplot of quantitative data (angle, torque, and pressure) from SBG (student #3) in **(A)** pretest and **(B)** posttest; **(C)** comparison result of statistical analysis of parameters as angle, torque, angular velocity, pressure, and pain level; **(D)** comparison result between SAG (graph-based) and SBG (visual-based). *p 0.05 and **p 0.001.

Finally, we analyzed the testing results of CaTARo with 3D facial expression using two students (SBG). Figure 10A,B show an example of 3D scatterplots of the angle (*x*-axis), torque (*y*-axis), and pressure (*z*-axis) data for the pretest and posttest from one subject (SBG-student #3). In the pretest (Figure 10A), the angle and torque pattern of CaTARo were not constant during the ROM exercise. In particular, the pressure (*z*-axis) value was found to be around 1,000 and 1,500. Conversely, in the posttest (Figure 10B), the angle and torque pattern of CaTARo were constant, and the *z*-axis values indicating the pressure appears to be zero.

Figure 10C shows a comparison result of statistical analysis of parameters as the angle, torque, angular velocity, pressure, and pain level in pre- and posttest from SBG. It is evident that the values of all parameters, except angular velocity, have decreased, and it was confirmed that the pain level decreased by roughly 2.37 in the post-evaluation rather than the pre-evaluation. This decrease in the parameter values in the posttest can be inferred as a positive aspect in which students performed care education while observing the range of movement of CaTARo after 30 min of care training. In particular, the parameter of pressure (M = 16.63, SD = 46.24) in the post-evaluation of SBG showed a relatively low value and decreased by about 429.81 compared to that of pressure in pretest, thereby indicating the effectiveness of the results.

Finally, Figure 10D presents a comparison of the pain-level results between SAG (graph-based) and SBG (visual-based). There was no significant difference in the post-evaluation (*t* = 6.043, *p* = 0.052) of SAG (M = 4.57, SD = 2.92) and SBG (M = 2.92, SD = 2.31). Compared to the pretest, the pain level of SAG (*t* = 21.073, *p* < 0.001) and SBG (*t* = 32.961, *p* < 0.001) decreased by 1.63 and 2.32 in the posttest, respectively. Therefore, we can infer that CaTARo with the 3D facial pain expression system can allow students to receive feedback on their care training in real time and help improve their care skills.

### 5.3 Questionnaire

Last, we performed a survey with the five elderly care experts and two students regarding the proposed CaTARo system. The survey for experts consisted of the following four questions:

• Q1: Does the CaTARo system with the real-time monitoring program provide a user-friendly interface?
• Q2: Do you think the movements of the robot are similar to those of a real human?
• Q3: How satisfied are you with the CaTARo system for the training of new caregivers?
• Q4: Would you be willing to use the CaTARo system for elderly care training considering ROM exercises in the future?

Table 4 shows the results of the questionnaire from the five experts. Out of a maximum score of 5, the mean scores of Q1, Q2, Q3, and Q4 were 3.4 (±0.55), 2.6 (±0.89), 3.6 (±0.55), and 4.4 (±0.55), respectively (numbers in parentheses are standard deviations). These results are favorable, although the experts indicated that the perceived feel of the robot was slightly different from that of the movement of a human elbow. Therefore, we believe that the proposed CaTARo system will be helpful for trainees in the future due to its friendly interface and potential use in elderly care training.

**TABLE 4:** Questionnaire results from five experts.

	Q1	Q2	Q3	Q4
Expert 1	3	2	3	4
Expert 2	3	2	4	4
Expert 3	4	4	4	5
Expert 4	4	2	3	4
Expert 5	3	3	4	5
Total	3.4 (0.55)	2.6 (0.89)	3.6 (0.55)	4.4 (0.55)

The maximum score for each item is five points. The numbers in parentheses are standard deviations.

The survey for students consisted of the following three questions:

• Q5: Does the CaTARo system based on 3D avatars provide a user-friendly interface?
• Q6: Was there any difference between the CaTARo system with 3D avatars and the existing method?
• Q7: How satisfied are you with the CaTARo system for the training of students or novices?

Table 5 shows the results of the questionnaire from the two students. The mean scores of Q1, Q2, and Q3 were 4.3 (±0.4), 4, and 4.3 (±0.4) out of a maximum score of 5, respectively. These results are relatively favorable, but it is still difficult to generalize the conclusion because there were two subjects who participated in the questionnaire. However, the students indicated that the perceived feel of the robot with the 3D avatar was slightly different from that of the existing system without the 3D avatar.

**TABLE 5:** Questionnaire results from two students.

	Q5	Q6	Q7
Subject 3	4	4	4.5
Subject 4	4.5	4	4
Total	4.3 (0.4)	4 (−)	4.3 (0.4)

The maximum score for each item is five points. The numbers in parentheses are standard deviations.

### 5.4 Limitations

This study examined the possibility and feasibility of using a robot with 3D avatar in care training. The pilot test and results of our study may serve as motivation and inspiration for the necessity of an effective care education robot that can provide feedback. However, this study has a limitation: the number of participants was not sufficient to draw generalized conclusions. In addition, although only the torque value of the experts in our experiment showed significant differences, for an optimal evaluation, the numbers of participants in the two groups must be equal. Hence, additional participants will need to be recruited such that more experiments can be performed to generalize the obtained findings in a future study. Finally, the proposed pain-level measurement method allowed for the determination of PSPI based on the shoulder range of motion. A more robust method to identify the correct pain group will be investigated in a future study by obtaining PSPI values from facial images acquired from tests of the elbow range of motion.

## 6 Conclusion and Future Works

We presented a care training assistant robot with a 3D facial pain expression system for use in accurate and efficient elderly care training. First, we developed an upper-limb robotic simulator that can improve the effectiveness of training for the care and treatment of the elderly, and proposed a method to obtain the pain level based on fuzzy logic. In addition, we developed a 3D facial pain expression avatar that can provide feedback to students more efficiently when using CaTARo. Experiments were conducted with five elderly care experts and four students who performed a range of motion exercises using CaTARo. We obtained quantitative data from these tests, including the elbow joint angle, torque, and pressure values from the robot. We found that the quantitative data results between experts and trainees differed, especially before training. Finally, we conducted a survey questionnaire with the elderly care experts and students concerning the effectiveness and feasibility of the proposed system; this survey obtained positive feedback.

To enhance the quality of the proposed system, we will continue to investigate the proposed elderly care training system to apply these functions and will improve a 3D avatar for the shoulder complex joint, which was developed in a previous study, to conduct a passive range of motion experiment.

## Data Availability

The raw data supporting the conclusions of this article will be made available by the authors, without undue reservation.

## Abbreviations

CaTARo, care training assistant robot; ROM, range of motion; PG, pain group; SMA, signal magnitude of area; FIS, fuzzy inference system; AU, action unit; PSPI, Prkachin and Solomon Pain Intensity Scale; EG, expert group; SAG, student experimental group A (CaTARo with graph-based feedback); SBG, student experimental group B (CaTARo with visual-based feedback); IRB, institutional review board; M, mean; SD, standard deviation.
